# Supplementary material for: Exploring perceptions of and attitudes towards tanning with school children, parents/carers and educators in Wales: A mixed methods study protocol for the SunChat study
Source: PLoS One. 2024 Jun 5;19(6):e0295719. doi: 10.1371/journal.pone.0295719 (PMC11152271; doi:10.1371/journal.pone.0295719)
Supplement: S1 Appendix — (DOCX) [file pone.0295719.s001.docx]

**Sun-Chat: SUN safety Conversations about Healthy Attitudes to Tanning: exploring perceptions of school children and their parents/carers**

**Activity 1**

**Sun-tanning perceptions**

| **Brief activity overview** | **Resources needed (per school):** |
| --- | --- |
| Brief activity is designed to provide an insight into how children perceive sun-tanning.  This activity should take approximately 10-15 minutes. | Materials   - 2-pack skin-colour crayons (similar to scale ‘human skin colour’ including red and pink options) - Child-figures to colour (2 per child)   Technological devices   - 1 audio recorder (check charge/batteries) |

**Activity instructions**

Step 1 – Setting up the space:

- Identify children who will take part in the activity and go to a separate room or stay in the same classroom depending on the teacher’s preference.
- Ensure all documentation (e.g., information sheet/consent) has been completed and agreed.
- Child consent and introduce the scribe
- Let children know they may come in and out of the activity if they need to go to toilet, concentration break, etc.
- Place materials in the centre of the table for children to access easily.
- Place the audio recorder in the centre of the table and begin recording.

Step 2 – Starting the activity (5 mins):

Read to the children: *Today we’ll do an activity together. We want to know more about activities you do in the summer. What do you like to do on holidays (here or abroad)?*

*Do you like to do activities in the sun? Go to the beach? Go to the park? Play outdoors? Engage children to answer the questions.*

*Our child here (talking about the figure) also enjoys playing and having fun in the sun.*

*Now, we will colour our child (they can name the picture) using the ‘skin-colour’ crayons (as you can see, we have different colours here).*

*First, you will be choosing a ‘skin colour-crayon’ and you’ll colour your child with any skin colour you like.*

*Our little friend is now on holidays and the weather is nice; they love playing in the sun.*

*Do you think their skin colour will keep the same after playing in the sun or will change? Yes/No? Why?*

*Let’s colour now our friend after enjoying in the sun; you can choose again a skin colour-crayon; it can be the same colour or a different one if you think our friend skin colour has changed.*

*Give them figure 2 and invite them to colour the child.*

*We will be here to help you with this– does this all sound okay?*

*Let’s get started!*

Step 3 – During activity (5-10 mins):

- The researcher assists children
- Continuously check children are comfortable through body language, if any children look uncomfortable, check if they are fine to continue and remind them their right to withdraw.
- During the activity ensure children are colouring the child-figure:

Prompt questions:

- *What thoughts and feelings are you having about this activity?*

*- What kinds of things would the child be doing in the sun?*

*- Do they like being in the sun*? *Why? Can you think of a few reasons?*

*- Can you think of any photos or other children you have seen enjoying the sun or after a holiday? What stands out for you about their skin?*

Step 4: After activity (10):

- When activity time is up, explain to children you will now be looking at each other’s children.

Example prompt questions to understand children’s perceptions of tanning:

- *How our children look before playing on the beach, the park..., on a summer day?*
- *Show drawings to each other*
- *And how they look after, when they are back home, indoors?*
- *Show drawings to each other*
- *Is there any difference? Do they look the same?*
- *Why did you choose this colour (same or different)?*
- *Which child’s skin colour do you prefer? (Make it clear that ‘skin’ colour is related to the sun, ‘before/after’)*
- *Is your child tanned?*
- *Do they look pretty? (Show them the first figure; and then the second figure) Depending on answers, why do they look pretty? Why don’t they look pretty/healthier? (Depending on the answer)*
- *Who looks healthier? (Show them the first figure; and then the second figure) Depending on answers, why do they look healthier?*
- *Does your child (dis-)like to be tanned? Do you know why?*
- *Is there anything you (dis-)like about tanning? What? Why?*
- Start engaging children with effects of sun-tanning on their health for activity 2, seeing what their preconceptions are.
- Link with activity 2 to understand what healthy habits they already know
