# Supplementary material for: Exploring perceptions of and attitudes towards tanning with school children, parents/carers and educators in Wales: A mixed methods study protocol for the SunChat study
Source: PLoS One. 2024 Jun 5;19(6):e0295719. doi: 10.1371/journal.pone.0295719 (PMC11152271; doi:10.1371/journal.pone.0295719)
Supplement: S2 Appendix — (DOCX) [file pone.0295719.s002.docx]

**SunChat: SUN safety Conversations about Healthy Attitudes to Tanning: exploring perceptions of school children and their parents/carers**

**Activity 2**

**‘Book of favourite activities in the sun’**

| **For researchers: brief activity overview** | **Activity resources needed* (per school):** |
| --- | --- |
| This activity is designed to understand existing healthy habits of children for sun-safety.  This activity should take approximately 25-30 minutes. | Materials   - 10 sheets of A1 flipchart paper - 1 pack of multicolour felt tip pens - 1 pack of colouring pencils - 1 pack of multicolour crayons - 10 glue sticks (1 per child) - Photos (with a range of objects, e.g., sunhat)   Technological devices   - 1 audio recorder (check charge/batteries) |

**Activity instructions**

Step 1 – Setting up the space and starting the activity (10 min.):

- Ensure you remind children that if they change their mind and are not happy to continue participating, that’s fine.
- Place 1 sheet of paper, 1 glue stick per child and the remaining materials in the centre of the table for children to access easily.
- Place the audio recorder in the centre of the table and verbally remind children of intention to record.

Read to the children: *For our next activity we will be drawing a picture of ourselves and our family doing our favourite activities in the sun.* *You can also choose some of the photos on the table (with a range of objects, e.g., sunhat) to glue in your drawing (collage).*

*Then, we will create our book of favourite activities in the sun!*

- Children drew a picture of themselves and their families doing their favourite activities in the sun (they can draw and use prompt pictures to identify if there are healthy habits in place).

*We will be here to help you with this– does this all sound okay?*

*Let’s get started!*

- The researcher assists children
- During the activity ensure children are fully focusing on their favourite activities in the sun
- Prompt questions:
- *What do you love doing in the sun?*
- *What do you need to enjoy the sun safely?*
- *Any special clothing? What else can you think about?*
- *What do you think happens to our skin when we are out in the sun?*
- *How long do you think you need to be in the sun before your skin changes?*
- *Is there a reason you would like to be in the sun on purpose for your skin to change?*

Step 2 – Watching a video[1] (5 mins):

Step 3 – After watching the video (10 mins):

Give children the time to consider if they would like to add something in their drawings after watching the video.

They can draw or use the pictures to protect themselves and their families from the sun.

When all children are ready and happy with their drawings, the researcher will create a cover page and assemble all drawings into a book of favourite activities in the sun.

Step 4: After activity (10 mins):

Follow with a discussion about what they have decided to include and why.

- When activity time is up, explain to children you will now be discussing what they put.

Prompt questions (these questions can be asked to the group, but we can also consider one to one conversation if children feel more confident to engage with the conversation):

*- What did you draw?*

*- Why did you include X?*

*-Have you added anything to your draw after watching the video? Why?*

*(Record/take notes of any changes and reasons why children change the draw)*

*- Do you have anything else to say or add?*

- We create our book of favourite activities in the sun.
- Thank children and let them have a break.

Reference:

1. Skcin, the Karen Clifford Skin Cancer Charity. George The Sun Safe Superstar- Book Animation, available from: https://www.youtube.com/watch?v=T7ghJsZug60, last accessed 25.03.24.
