# Supplementary material for: Exploring perceptions of and attitudes towards tanning with school children, parents/carers and educators in Wales: A mixed methods study protocol for the SunChat study
Source: PLoS One. 2024 Jun 5;19(6):e0295719. doi: 10.1371/journal.pone.0295719 (PMC11152271; doi:10.1371/journal.pone.0295719)
Supplement: S3 Appendix — (DOCX) [file pone.0295719.s003.docx]

**Sun-Chat: SUN safety Conversations about Healthy Attitudes to Tanning: exploring perceptions of school children and their parents/carers**

**Activity 3**

**Poster (co-designed with children for Schools)**

| **For researchers: brief activity overview** | **Activity resources needed* (per school):** |
| --- | --- |
| This activity is designed to meet the overall aim of encouraging healthy behaviours and to promote sun safety.  A poster will be used to showcase schools’ and children involvement.  This activity should take approximately 25-30 minutes. | Materials   - 5 pieces A3 paper (1 per 2-3 children) - A1 papers - Clear contact paper - 10 glue sticks (1 per child) - 10 pencils (1 per child) - 10 kid safe scissors (1 per child) - Magazines - 1 pack of multicolour felt tip pens - 1 pack of colouring pencils   Technological devices   - 1 audio recorder (check charge/batteries) |

**Activity instructions**

Step 1 – Setting up the space:

- Children return following a break.
- Ensure you remind children that if they change their mind and are not happy to continue participating, that’s fine.
- Place the audio recorder in the centre of the table (verbal reminder about recording) and begin recording.
- Place 1 piece of A3 paper, 1 glue stick, 1 pencil and 1 pair of scissors, and the remaining materials in the centre of the table.
- Prepare children for the final activity and poster

Step 2 – Starting the activity (5 mins):

Read to the children: *For our final activity you will be helping other children to promote sun* *safety and healthy habits and learn why it is healthier not to tan on purpose. To do this we will create a big Poster!* *As before, we will be here to help you with this– does this all sound okay?*

- Invite children to cut out pictures from magazines or pick some available images of sun safe/healthy behaviours or materials, e.g., hats, umbrellas, sun protection that will help other children enjoying their favourite activities in the sun.
- We can divide the poster in two columns, one with YES (healthy/safe) and the other one with NO (unhealthy/unsafe) and let children select images from the magazines related to healthy and unhealthy habits; sun safety and unsafe behaviours and let them decide what goes in each column based on activity 2 discussion. The researcher can have some cut outs ready to stimulate the discussion and decision making, e.g., children wearing hats (to represent healthy habits) or people tanning (to represent unhealthy behaviours).
- You will work together in group of 2-3 to make a small poster and then we will put all the creations together to make one large Poster.
- You need to place the images under each column YES (healthy/safe) or NO (unhealthy/unsafe).

Step 3 – During activity (15 mins):

- The researcher assists children and discuss with them healthy/unhealthy habits; bring points of activity 2 discussion.
- During the activity get them to think about sun safety and healthy behaviours.
- When children place images under each column in the Poster, make sure children are thinking about all things in relation to sun-safety and healthy habits.

Step 4: After activity (10-15 mins):

- When activity time is up, explain to children you will now be discussing what they put.

Prompt questions:

- *Ask children about their pictures and drawings on the poster.*
- *Why have they chosen these pictures?*
- *Why is important to wear a hat, sun protection, etc….?*
- *What have they chosen as unhealthy/unsafe behaviours and why?*
- *What would you say to other children to help them protecting from the sun and enjoy safely?*
- *What would you say to other children about unhealthy habits?*
- *Do you think you have changed your mind about tanning today?*
- Cover the poster board with contact paper to create a big Poster
- Thank the children and let them head back to their peers/classroom.
- Stop the audio recorder, take pictures of the Poster
- Write field notes and upload on Teams
