# Supplementary material for: Exploring perceptions of and attitudes towards tanning with school children, parents/carers and educators in Wales: A mixed methods study protocol for the SunChat study
Source: PLoS One. 2024 Jun 5;19(6):e0295719. doi: 10.1371/journal.pone.0295719 (PMC11152271; doi:10.1371/journal.pone.0295719)
Supplement: S4 Appendix — (DOCX) [file pone.0295719.s004.docx]

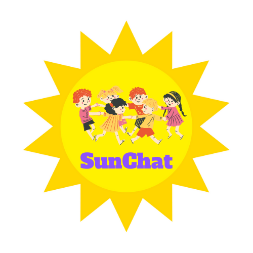


# SunChat: exploring perceptions of school children and their parents/carers

## Online Survey

Thank you for agreeing to complete this brief survey!

The questions should take less than **10** minutes to complete, and your answers will help us better understand what parents think of sun tanning both for themselves and their children.

All answers are confidential, and we will report all results anonymously so no one will be able to see which answers are yours.

You have the right to close the survey at any time and your answers will not be saved.

For more information about the study, please click here to download the Information Sheet. [Link to SunChat Online Survey Information Sheet and Consent Form V1 01.02.23] and for any questions regarding use of the survey, please contact [k.j.lanyon@swansea.ac.uk](mailto:k.j.lanyon@swansea.ac.uk).

Please aim to answer all questions to the best of your knowledge and honestly, there will be no judgement or consequences to any of your answers.

Thank you in advance for your time!

***Section 1 About you***

**Q1. Are you a parent of at least one child of primary school age?** Format: Single choice MANDATORY

|  | YES |
| --- | --- |
|  | NO |

*Message if no: Unfortunately, you are not eligible to take part if you are not a parent of a child of primary school age. Thank you for your time and interest in our research. Please feel free to send this link to any primary school aged parents you know. [STOP survey]*

**Please answer the following survey for your eldest child of primary school age only.** If you have one child only, please answer the following questions in relation to them.

**Q2. Where do you live?:** Format: single choice MANDATORY

|  | Wales |
| --- | --- |
|  | England |
|  | Scotland |
|  | Northern Ireland |
|  | Republic of Ireland |
|  | Prefer not to say |
|  | Other (please specify in the space below) |

**Q2a. Other, please specify:** Format: Free text box

|  |
| --- |

**Q3. What is your employment status?:** Format: Single choice MANDATORY

|  | Full-time employee |
| --- | --- |
|  | Part-time employee |
|  | Self employed |
|  | Student |
|  | Temporarily unemployed |
|  | Unemployed due to disability |
|  | Retired |
|  | Prefer not to say |
|  | Other (please specify in the space below) |

**Q3a. Other, please specify:** Format: Free text box

|  |
| --- |

**Q3b. What is your job role?:** Format: free text box (appears only if full-time, part-time or self-employed selected)

|  |
| --- |

**Q4. What is your child’s gender?:** Format: Single choice

|  | Female |
| --- | --- |
|  | Male |
|  | Other gender |
|  | Prefer not to say |

**Q5. What year is your child in?:** Format: Single choice

|  | Nursery |
| --- | --- |
|  | Reception |
|  | Year 1 |
|  | Year 2 |
|  | Year 3 |
|  | Year 4 |
|  | Year 5 |
|  | Year 6 |

**Q6.** Look at the scale below:


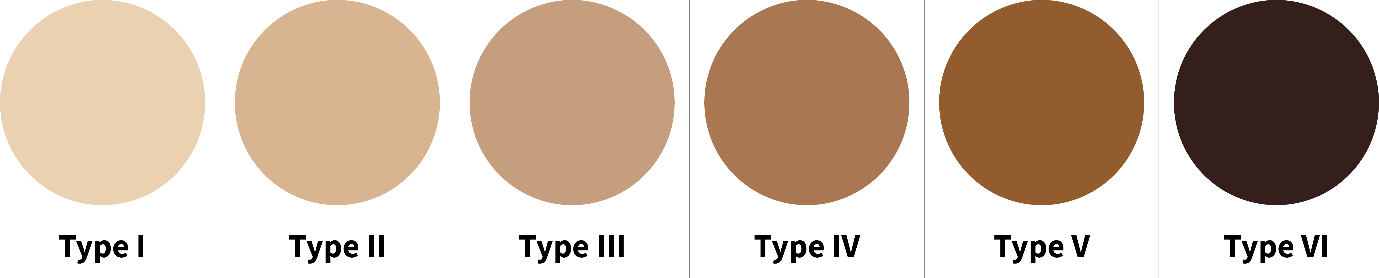


**What type most closely represents the colour of your child’s untanned skin?** Format: Single choice

|  | Type I |
| --- | --- |
|  | Type II |
|  | Type III |
|  | Type IV |
|  | Type V |
|  | Type VI |

This question uses a visual colour scale based on the Fitzpatrick scale.

**Q7. How old are you?** Format: Free text (with validation for whole number only, max 3 digits)

|  | Years |
| --- | --- |

**Q8. What is your gender?** Format: Single choice

|  | Female |
| --- | --- |
|  | Male |
|  | Other |
|  | Prefer not to say |

**Q9.** Look at the scale below:


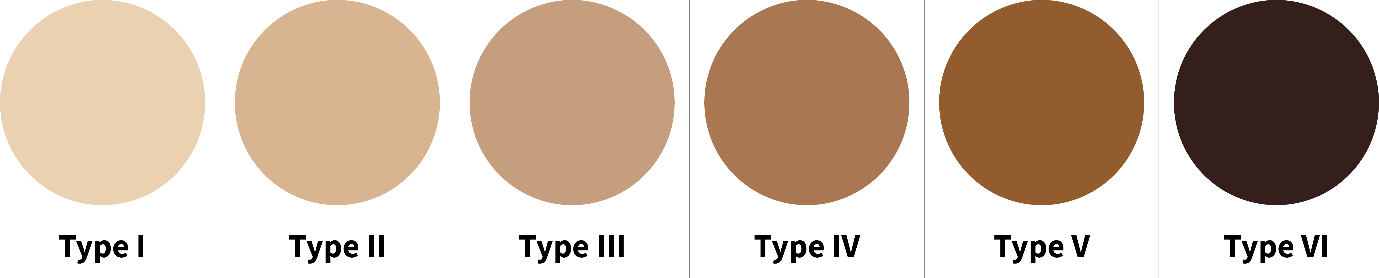


**What type most closely represents the colour of your untanned skin?** Format: Single choice

|  | Type I |
| --- | --- |
|  | Type II |
|  | Type III |
|  | Type IV |
|  | Type V |
|  | Type VI |

This question uses a visual colour scale based on the Fitzpatrick scale.

***Section 2: Your thoughts about sun tanning***

Please indicate your agreement or disagreement with the following statements. Please feel free to record any additional comments you have.

**Q10. I feel more attractive with a suntan.** Format: Single choice

|  | Strongly agree |
| --- | --- |
|  | Somewhat agree |
|  | Neither agree nor disagree |
|  | Somewhat disagree |
|  | Strongly disagree |
|  | Don’t know/prefer not to answer |

**Comments:**

**Q11. I feel healthier with a suntan.** Format: Single choice

|  | Strongly agree |
| --- | --- |
|  | Somewhat agree |
|  | Neither agree nor disagree |
|  | Somewhat disagree |
|  | Strongly disagree |
|  | Don’t know/prefer not to answer |

**Comments:**

**Q12. Sun tanning is necessary for my body to produce Vitamin D**. Format: Single choice

|  | Strongly agree |
| --- | --- |
|  | Somewhat agree |
|  | Neither agree nor disagree |
|  | Somewhat disagree |
|  | Strongly disagree |
|  | Don’t know/prefer not to answer |

**Comments:**

**Q13. There are no risks involved when tanning through sun exposure.**  Format: Single choice

|  | Strongly agree |
| --- | --- |
|  | Somewhat agree |
|  | Neither agree nor disagree |
|  | Somewhat disagree |
|  | Strongly disagree |
|  | Don’t know/prefer not to answer |

**Comments:**

**Q14. I feel it is worth getting sunburnt so that I can get suntanned.** Format: Single choice

|  | Strongly agree |
| --- | --- |
|  | Somewhat agree |
|  | Neither agree nor disagree |
|  | Somewhat disagree |
|  | Strongly disagree |
|  | Don’t know/prefer not to answer |

**Comments:**

**Q15. A suntanned child looks healthier than one without a suntan.** Format: Single choice

|  | Strongly agree |
| --- | --- |
|  | Somewhat agree |
|  | Neither agree nor disagree |
|  | Somewhat disagree |
|  | Strongly disagree |
|  | Don’t know/prefer not to answer |

**Comments:**

**Q16. Sun tanning is necessary for my child to produce Vitamin D**. Format: Single choice

|  | Strongly agree |
| --- | --- |
|  | Somewhat agree |
|  | Neither agree nor disagree |
|  | Somewhat disagree |
|  | Strongly disagree |
|  | Don’t know/prefer not to answer |

**Comments:**

**Q17. I am happy for my child to get a suntan.** Format: Single choice

|  | Strongly agree |
| --- | --- |
|  | Somewhat agree |
|  | Neither agree nor disagree |
|  | Somewhat disagree |
|  | Strongly disagree |
|  | Don’t know/prefer not to answer |

**Comments:**

**Q18. I worry about future sun damage to my child’s skin.** Format: Single choice

|  | Strongly agree |
| --- | --- |
|  | Somewhat agree |
|  | Neither agree nor disagree |
|  | Somewhat disagree |
|  | Strongly disagree |
|  | Don’t know/prefer not to answer |

**Comments:**

***Your children’s thoughts about suntanning***

Please indicate your agreement or disagreement with the following statements. Please feel free to record any additional comments you have. Format: single choice

**Q19. My child thinks that they look better with a tan.** Format: Single choice

|  | Strongly agree |
| --- | --- |
|  | Somewhat agree |
|  | Neither agree nor disagree |
|  | Somewhat disagree |
|  | Strongly disagree |
|  | Don’t know/prefer not to answer |

**Comments:**

**Q20. My child feels pressure from the social media, tv and video streaming platforms to have a tan.** Format: Single choice

|  | Strongly agree |
| --- | --- |
|  | Somewhat agree |
|  | Neither agree nor disagree |
|  | Somewhat disagree |
|  | Strongly disagree |
|  | Don’t know/prefer not to answer |

**Comments:**

**Q21. My child feels pressure from their peers to have a suntan.** Format: Single choice

|  | Strongly agree |
| --- | --- |
|  | Somewhat agree |
|  | Neither agree nor disagree |
|  | Somewhat disagree |
|  | Strongly disagree |
|  | Don’t know/prefer not to answer |

**Comments:**

**Q22. My child feels pressure from a sibling to have a suntan.** Format: Single choice

|  | Strongly agree |
| --- | --- |
|  | Somewhat agree |
|  | Neither agree nor disagree |
|  | Somewhat disagree |
|  | Strongly disagree |
|  | Not applicable/prefer not to answer |

**Comments:**

If respondent chooses “Strongly agree or Somewhat agree ” above, Q20a opens.

**Q22a. What is the gender of the sibling who mainly influences them?** Format: single choice

|  | Female - older |
| --- | --- |
|  | Female - younger |
|  | Male - older |
|  | Male - younger |
|  | Other gender |

**Comments:**

***Sun habits***

Please indicate your agreement or disagreement with the following statements. Please feel free to record any additional comments you have. Format: single choice

**Q23. I find it difficult** **to protect my child from the sun.** Format: single choice/free text

|  | Strongly agree |
| --- | --- |
|  | Somewhat agree |
|  | Neither agree nor disagree |
|  | Somewhat disagree |
|  | Strongly disagree |
|  | Don’t know/prefer not to answer |

**Comments:**

If respondent chooses “Strongly agree or Somewhat agree”, Q23a opens.

**Q23a. What is the reason why you feel it is difficult to protect your child from the sun?**

|  |
| --- |

**Q24. I intentionally sunbathe in order to get a tan (please select all that apply):** Format: Multiple choice/free text

|  | While on a holiday |
| --- | --- |
|  | Using UV tanning salons |
|  | While the weather is sunny |
|  | Never |
|  | Other |

**Comments:**

**Q24a. If you selected “other” please specify in the space provided.**

|  |
| --- |

**Q25. My child intentionally sunbathes in order to get a tan (please select all that apply):** Format: Multiple choice/free text

|  | While on a holiday |
| --- | --- |
|  | Using UV tanning salons |
|  | While the weather is sunny |
|  | Never |
|  | Other |

**Comments:**

**Q25a. If you selected “other” please specify in the space provided.**

|  |
| --- |

***Final comments***

**Do you have any other comments on anything we’ve asked here? Please share your feedback below.** Format: Free text

Please enter comments here:

|  |
| --- |

**Would you like to be informed of the results of this survey?** Format: Single choice

|  | YES |
| --- | --- |
|  | NO |

If yes, please enter your email address here:

|  |
| --- |
